# Game Changer: Unveiling the Significance of Game Design Elements as Encouragement Strategies during Maximal Exercise Testing

**DOI:** 10.1186/s40798-025-00922-w

**Published:** 2025-10-15

**Authors:** Sascha Ketelhut, Ralf Brand, Daniel Hug, Florian ‘Floyd’ Mueller, Anna Lisa Martin-Niedecken

**Affiliations:** 1 Institute of Sport Science, University of Bern, Bremgartenstrasse 145, Bern, 3012 Switzerland; 2 Sport and Exercise Psychology, University of Potsdam, Potsdam, Germany; 3 Zurich University of the Arts, Institute for Computer Music and Sound Technology, Zurich, Switzerland; 4 Exertion Games Lab, Department of Human-Centered Computing, Monash University, Melbourne, Australia; 5 Zurich University of the Arts, Institute for Design Research, Zurich, Switzerland

**Keywords:** Maximal exercise testing, Gamification, Encouragement strategies

## Abstract

Maximal exercise testing is a fundamental component of sports medicine and clinical practice, essential for evaluating physical fitness, tailoring training programs, and diagnosing health conditions. A crucial aspect of maximal exercise testing is ensuring that participants exert maximal effort, as insufficient effort can compromise the validity of results, potentially leading to misdiagnoses, misinterpretation of outcomes, and inappropriate exercise recommendations. Various strategies, including verbal, audio, and video-based methods, have been used in research and practice to encourage maximal effort. Despite the recognized importance of these strategies, understanding of them remains limited, with recommendations being either inconsistent or entirely lacking. Notably, innovative approaches that harness the potential of digital methods are still relatively scarce. In this article, we discuss the potential of incorporating game elements as an innovative encouragement strategy during maximal exercise testing. Drawing from research on exergaming, we provide examples of impactful game features and discuss their potential integration into exercise testing. This innovative approach has the potential to improve test reliability, enhance validity, streamline workflows, and positively influence attitudes toward exercise testing. We advocate for establishing a new area of research focused on gamifying maximal exercise tests to elevate exercise diagnostics to the next level.

## Key Points

• Maximal exercise testing is an important tool for assessing physical fitness and diagnosing health conditions. A critical factor in ensuring the validity of these tests is confirming that participants exert maximal effort, which can be particularly challenging in certain target groups.
• Currently, verbal encouragement is predominantly used in research and practice. However, this is difficult to standardize and often lacks engagement, as it heavily depends on the individual delivering it. Game design elements may offer a more effective approach by fostering a dissociative focus, enhancing motivation, and increasing effort investment.
• Immersive and engaging game design elements used as encouragement strategies may improve the validity of maximal exercise tests and enhance compliance, especially in populations with lower motivation.

## Introduction

Maximal exercise testing is a critical diagnostic tool used to assess physical fitness, track progress, customize training programs, diagnose health conditions, and assist healthcare professionals in creating personalized treatment and rehabilitation strategies. A key factor for accurate maximal exercise testing is ensuring that participants exert their maximal effort, as insufficient effort can compromise the validity of the results [1]. To address this, effective encouragement strategies are essential to support participants in fully committing to the test [1, 2]. This perspective advocates for exploring the incorporation of game design elements as innovative encouragement strategies during maximal exercise testing to enhance the testing experience and improve test validity and reliability.

According to the dual mode theory, higher physical exhaustion during exercise leads to more negative affective responses due to the combined influence of physical sensations and cognitive appraisals [3]. As exercise intensity increases and reaches the ventilatory threshold, physiological stress and discomfort also increase, caused by muscle pain and breathlessness. These intense physical sensations are inherently aversive, resulting in negative affective responses. At moderate intensity levels, cognitive appraisals such as thoughts about health benefits or personal goals can help buffer negative feelings, but as intensity further increases beyond the ventilatory threshold, cognitive control diminishes. The overwhelming physical sensations then overshadow any positive cognitive appraisals, leading to a more negative overall experience [3]. For those who are unaccustomed to exercise, these sensations can be even more intense [4], potentially leading them to terminate maximal exercise tests well before reaching their physical limits [5]. Consequently, encouraging individuals to enhance commitment to effort investment during maximal exercise tests is crucial, especially within specific target groups such as inactive individuals, at-risk patients, children, and those with mental disabilities [1]. Without sustained motivation and concerted effort, there is a reduced likelihood that participants’ scores will reflect their true fitness levels, resulting in inaccurate performance assessments, misdiagnoses, and the implementation of inappropriate exercise and treatment strategies. Therefore, conducting maximal exercise testing in a manner that actively encourages individuals to exert maximal effort is imperative to enhance the internal validity of the test [6].

Various encouragement strategies, including verbal, audio, and video-based methods, have been employed in research and practice. These encouragement strategies can enhance performance by positively influencing core affect, mood, and self-efficacy [7–13]. Additionally, they can help redirect attentional focus, facilitating dissociation from the unpleasant sensations of exertion [7]. This dissociative effect likely occurs because the perception and processing of these stimuli compete for the limited cognitive capacity needed to perceive exertion [9]. Consequently, the perceptual load induced by encouragement strategies is considered a key modulating factor in their potential to facilitate dissociation [8]. Building on this premise, this Current Opinion advocates for a shift towards integrating game design elements as encouragement strategies for maximal exercise testing. We propose moving beyond traditional methods to explore and develop innovative, immersive, and highly engaging gamified approaches. These new strategies have the potential to enhance the overall testing experience, increase participants’ willingness to exert maximal effort, and advance standardization to improve test reliability.

## The Potential of Gamification

Research suggests that audio (music or sound effects) or visual (video) stimulation enhances affective responses during exercise and exercise testing by competing for attentional resources against interoceptive afferents generated by high-intensity exercise [9, 10]. According to Hutchinson et al. [8] the potential effectiveness of a dissociative strategy is influenced by its perceptual load. Following this principle, it is reasonable to anticipate that further intensifying the sensory stimulation during exercise would exceed the impact of the previously studied audio or video stimuli across various outcomes related to exercise experience. In this context, digital games or game elements may be promising as they impose an even greater perceptual load.

Unlike passive media such as videos or music, digital games combine complex visual and auditory stimulation with active engagement requiring the user to overcome challenges, process information, apply strategic elements, and rapidly respond to multiple cues simultaneously [14]. Furthermore, digital games inherently possess a high level of motivational potential [15], enhancing perceptions of autonomy, competence, and social-relatedness [16]. This is significant, as enhancing these perceptions supports self-efficacy for the task, which is critical for motivation [17]. Recognizing this potential, it has become highly popular to integrate game design elements, such as points, badges, leaderboards, meaningful stories, and avatars, into non-game environments, commonly referred to as gamification [18].

Gamification is widely acknowledged as an innovative and promising concept applicable across various contexts and target groups to motivate specific behavior and increase enjoyment [16]. Various test publishers and companies are already using gamification to develop robust psychometrics, enhancing the validity of assessments [19, 20]. Importantly, clear distinctions should be made between different uses of game-related approaches. Gamification refers to the integration of isolated game-like elements (e.g., points, badges, leaderboards) into non-game contexts. Game design, by contrast, encompasses broader aspects such as narrative structures, gameplay mechanics, and immersive worlds. Fully developed game genres, such as serious games or games designed explicitly for purposes beyond pure entertainment, build upon game design rather than gamification alone. Serious games can cover a wide range of domains – including movement-based genres such as exertion games (exergames). Surprisingly, neither gamification, nor game design, nor serious games have yet been applied in the context of maximal exercise testing. The integration of game design elements as a form of multisensory stimulation into maximal exercise testing therefore represents a novel and largely unexplored approach with significant potential to enhance the dissociative effect.

## Exergames as a Promising Example

Exergames combine physical and cognitive activities with immersive game experiences, requiring players to be physically engaged to control the game [21, 22]. A concrete example is the ExerCube, an innovative, immersive exergaming system that combines functional, high-intensity full-body movements with an interactive game environment projected onto three surrounding walls. The ExerCube continuously adapts game difficulty in real time based on the player’s in-game performance and heart rate, dynamically adjusting movement speed, cognitive task complexity, and physical challenge. This system enables individually tailored training intensities that can reach vigorous to maximal exertion levels. Immediate audiovisual feedback, clear goals, and reward mechanisms help sustain motivation and promote flow experiences, transforming the workout into an engaging, and highly personalized, game experience [23].

Research indicates that well-designed exergames may positively impact enjoyment [24, 25], motivation [26], engagement [27, 28], and mood [29]. Feedback, challenge, and rewards are mechanisms by which exergames can induce positive affective responses [27]. Additionally, owing to the core elements of gameplay and the rich and complex audiovisual environment that ensures immersion, exergames divert individuals from psychological cues, creating a more game-like and less exercise-like experience [30]. It has been shown that the highly engaging nature of exergames has the potential to facilitate a dissociative focus, enabling individuals to achieve higher exercise intensity before the perception of exertion becomes too prominent [31]. However, it must be noted that most of the current exergames studied in research only induce low to moderate exercise intensities [21]. Therefore, the transferability of the reported affective responses to higher exercise intensities or even maximal exercise tests remains uncertain. However, in a recent study, we \showed that specific exergames can attain high-intensity levels, with a mean heart rate of up to 86% of maximum, a peak heart rate of 97% of the maximum heart rate, and a mean metabolic equivalent of task of 9.2 [26, 32]. The perceived enjoyment, flow experience, and intrinsic motivation were significantly higher during the high-intensity exergaming session than during a moderate endurance exercise [24, 26]. Interestingly, age, sex, body mass index, and physical fitness showed no significant moderating effect on the exercise intensity achieved. This suggests that specific demographic or physiological variables, which are typically reported to influence the willingness to invest effort during physical exercise [33], are not relevant in the context of exergaming. Moreover, participants in this study reported higher enjoyment during the exergaming session that exceeded the anaerobic threshold compared to endurance exercise below the anaerobic threshold. The study further observed that 14% of the participants achieved higher heart rates and oxygen uptake than during a cardiopulmonary exercise test on a treadmill. This suggests a greater willingness to exert effort during the exergame compared to a standardized maximal exercise test with verbal encouragement.

It seems that the immersive and engaging nature of exergaming can effectively shift the player’s attention from an intrinsic focus to an external, game-directed focus. Therefore, it can be hypothesized that the incorporation of audio-visual elements into maximal exercise tests may yield comparable dissociative and motivational effects.

Although existing evidence suggests that exergaming can elicit high-intensity levels, there is still a lack of controlled studies directly comparing the effects of traditional encouragement strategies (e.g., verbal encouragement) with specific game design elements. Future research should address this gap by conducting randomized crossover studies in which participants perform maximal exercise tests under both classical and game-based conditions. To identify which game design components are most effective, such studies should systematically vary individual elements - such as feedback, rewards, and competition - to assess their specific and combined effects on performance and motivation.

## Gamifying Maximal Exercise Testing

As the validity of maximal exercise testing relies heavily on the willingness of an individual to exert maximal effort, it is crucial to create a test environment that encourages participants to continue the test when fatigue sets in and to alleviate negative perceptions associated with exertion. Incorporating game elements through audiovisual stimuli could transform the test from a monotonous task into an engaging and enjoyable experience. The competitive and goal-oriented nature of games can encourage individuals to push their limits and achieve in-game objectives, potentially enhancing the validity of the test. Additionally, gamified encouragement may provide a more standardized approach compared to traditional methods, such as verbal prompts, which strongly rely on the engagement of the test personnel, thus improving the test’s reliability. Furthermore, such an approach could alleviate the burden on test personnel, as they would not need to concentrate on providing encouragement. However, it is important to note that the final output is unlikely to be a fully developed video game, as this could compromise both the scientific validity of the test and the safety of participants. Instead, the focus should be on incorporating specific game design elements that enhance the testing environment and encourage greater effort and engagement.

Currently, there is a significant gap in our understanding of which game elements could be effectively utilized for maximal exercise testing. However, insights from exergaming research provide valuable guidance for the initial selection and methodological development of promising game elements [34]. Leveraging established exergame design frameworks [35–37] can also support the systematic and effective integration of gamification elements. A user-centered, iterative design approach is recommended, encouraging interdisciplinary collaboration among clinicians, exercise physiologists, psychologists, and game and sound designers throughout the development process. Table 1 provides examples of impactful game features and how they could be integrated into maximal exercise testing.

**Table 1:** Examples of game design elements and their potential implementation in maximal exercise testing

Design Elements	Description	Possible implementation
Incorporate Challenging Tasks	Design tasks that present users with meaningful challenges by integrating complex mechanics, strategic elements, and intuitive rules to keep users engaged and motivated.	**Virtual Opponents**: Incorporate digital opponents that dynamically adjust their performance to match the test’s level of challenge, creating a more competitive environment.
Provide Prompt Feedback	Ensure users receive immediate and clear feedback on their performance. This real-time information aids in tracking progress and maintaining motivation.	**Instantaneous Metrics**: Provide on-screen metrics like speed, power output, distance, and heart rate to inform users of their performance. **Messages**: Display encouraging messages or virtual trainers/spectators providing feedback as users reach milestones or struggle, reinforcing a supportive environment.
Utilize Narrative Structure	Develop a compelling narrative or context that adds emotional depth and provides a sense of purpose throughout the exercise task.	**Epic Journey**: Create a narrative where users are embarking on a journey through different worlds, with each stage representing a new environment or challenge. **Mission Objectives**: Assign mission-based tasks that align with the test challenges to create a goal-oriented approach. **Character Progression**: Allow users to customize avatars that evolve as they progress through the test, reflecting personal growth. **Interactive Storylines**: Allow users to make choices that affect the narrative outcome, keeping them engaged.
Foster Immersion	Achieve multiple forms of immersion to deeply engage users.	**Virtual Environments**: Use high-definition screens to create immersive, and engaging environments. **Audio Enhancements**: Implement adaptive surround sound with dynamic soundscapes that change according to the test’s challenges. **Progressive Challenges**: Introduce dynamically changing environments or scenarios that adapt to users’ performance.
Encourage Interactive Involvement	Design features that require active participation. Integrate interactive elements and feedback mechanisms to sustain engagement.	**Response-Based Tasks**: Integrate tasks where users must react to virtual stimuli (e.g., dodging obstacles) by increasing speed, power output, or adjusting intensity.
Implement Dynamic Rewards	Incorporate systems such as high scores, points, leaderboards, and badges to provide tangible goals and rewards. Dynamic rewards can help maintain a sense of achievement.	**Point Accumulation**: Award points for achieving specific heart rate zones, maintaining speed, or reaching distance milestones during each stage of the test. **Achievement Badges**: Provide badges for completing stages, achieving personal bests, or demonstrating consistent improvement over time. **Leaderboard Integration**: Allow users to see where they rank locally or globally against others who have taken the same test, encouraging friendly competition **Virtual Currency**: Introduce virtual currency earned by performance that can be used to unlock new features or accessories for avatars.
Ensure User Control	Allow users to feel a sense of control over their actions within the test/game. Providing autonomy enhances user satisfaction and encourages continued participation.	**Environment Selection**: Provide options to choose and easily switch different virtual environments, to keep the experience fresh and engaging. **Scenario Adjustments**: Allow users to customize virtual scenarios, such as adjusting weather conditions or time of day, to match their preferences and comfort levels.
Set Clear Goals	Define clear, achievable goals at appropriate stages. Clear objectives help users stay focused and maintain motivation.	**Progress Tracking Dashboards**: Display user progress on a dashboard that highlights completed stages, time remaining, and overall performance metrics. **Goal Completion Notifications**: Use notifications to alert users when they have achieved set goals (reaching standard values), enhancing the sense of accomplishment.
Facilitate Social Interaction	Create opportunities for competition and cooperation to enhance social interaction and increase engagement.	**Virtual Competitions**: Host online challenges where users can compete (alone or in teams) against others in real-time or asynchronously, with results shared on leaderboards. **Peer Support Networks**: Facilitate social networking by enabling users to connect with peers, share progress, and encourage each other through integrated chat systems or forums **Social Media Sharing**: Offer options to share achievements, badges, or progress updates on social media platforms to garner external support and recognition.

**Fig. 1:**
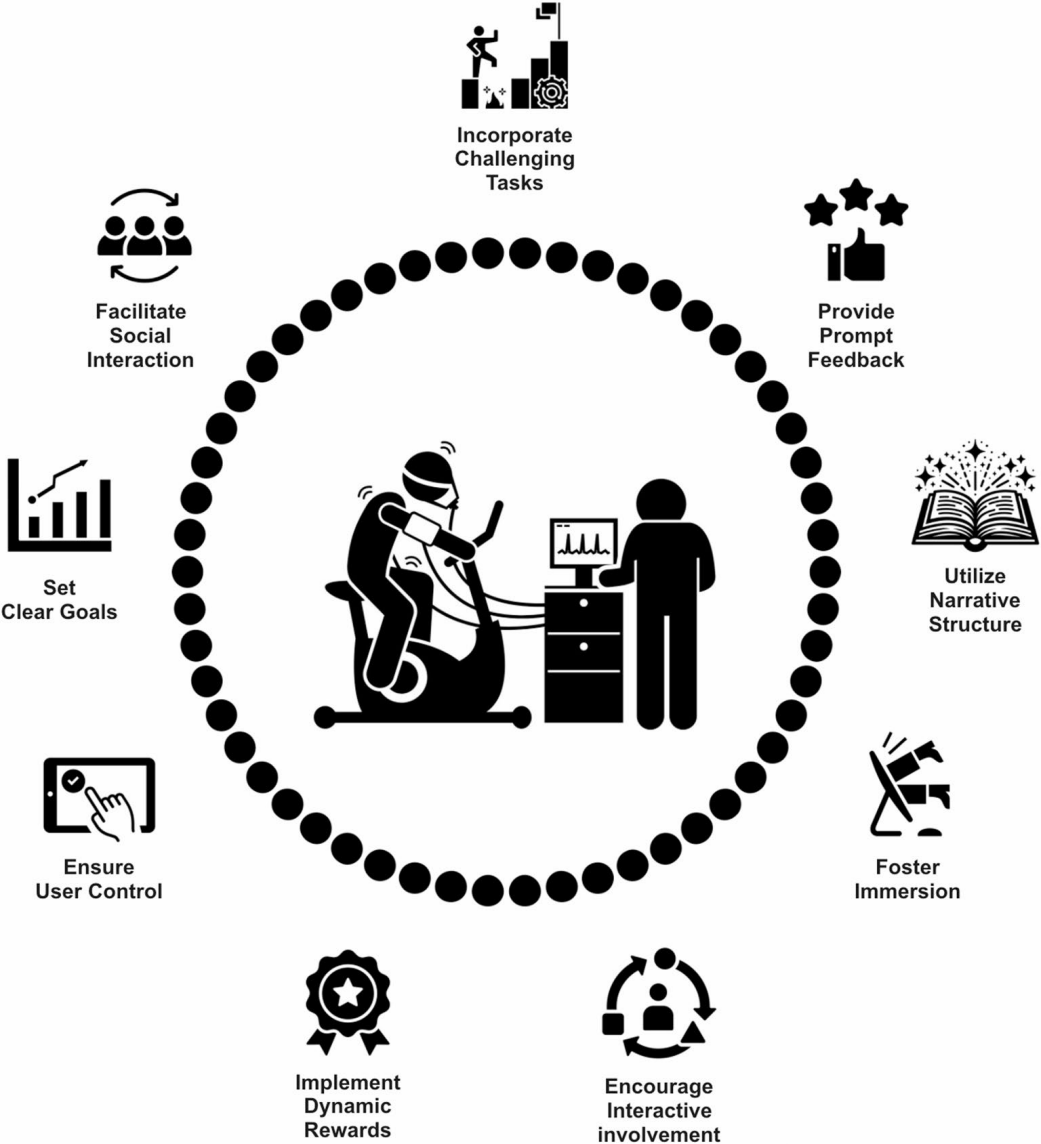
Examples of Game Design Elements in Maximal Exercise Testing

## Conclusion

The incorporation of game design into maximal exercise testing (Fig. 1) represents a transformative approach to motivate individuals to enhance their commitment to effort investment, an essential factor for test validity. This approach can streamline workflows during testing and help standardize encouragement, thereby positively affecting test reliability. By incorporating game elements and interactive audio-visual stimuli, the traditionally mundane testing process can be transformed into an engaging and more enjoyable activity. This may have the potential to shape participants’ attitudes toward exercise testing and physical activity in general [31, 38].

We advocate for establishing a new area of research focused on identifying effective game elements that potentially support maximal exercise tests while preserving scientific integrity and safety. This pioneering approach has the potential to revolutionize exercise diagnostics in both sports science and clinical practice.

## Data Availability

N/A.
